# Supplementary material for: Empirical methods for controlling false positives and estimating confidence in ChIP-Seq peaks
Source: BMC Bioinformatics. 2008 Dec 5;9:523. doi: 10.1186/1471-2105-9-523 (PMC2628906; doi:10.1186/1471-2105-9-523)
Supplement: Additional File 1 — A variety of html documents from the USeq web site detailing the available applications, their best usage, output file type descriptions, command line menus, etc. [file 1471-2105-9-523-S1.zip › USeqUserGuides/JavaDoc.zip!/JavaDoc/trans/tpmap/TPMapOligoBlastFilter.html]

TPMapOligoBlastFilter


|  |  |  |
| --- | --- | --- |
| **Overview** \| **Package** \| **Class** \| **Use** \| **Tree** \| **Deprecated** \| **Index** \| **Help** | |  |
| **PREV CLASS**   **NEXT CLASS** | **FRAMES**    **NO FRAMES**     **All Classes** |  |
| SUMMARY: NESTED \| FIELD \| CONSTR \| METHOD | DETAIL: FIELD \| CONSTR \| METHOD |  |


---


## trans.tpmap Class TPMapOligoBlastFilter

```
java.lang.Object
  trans.tpmap.TPMapOligoBlastFilter
```

---

``` public class TPMapOligoBlastFilter extends java.lang.Object ```

Application for remapping and filtering a text version bpmap file with BLAST.
This is wickedly slow :( ! Can't use -B or megablast so stuck with this junk. If you don't care about
mismatches then use the MummerFilter applicaiton.
Filters a tpmap file only printing out oligo lines that have one unique match to the sequenced genome.
The orientation direction is replaced with the number of 1bp matches for files appended with Bls or
total number of matches (1bp + exact) for files appended with NoTrmBls.
To launch on Sapo cluster: java -Xmx1500M nix/util/gen/JQSub cd /home/sapo/nix/NixSourceCode/ return java -Xmx1500M nix/affy/TPMapOligoBlastFilter -d /home/sapo/nix/Affy/BLAST/whole\_genome.fasta -s /home/sapo/software/seqanal/alignment/ncbi/bin/blastall -b /home/sapo/nix/Affy/TPMap/Extra/extra.tpmap

---

**Constructor Summary**

`TPMapOligoBlastFilter(java.lang.String[] args)`


| **Method Summary** | |
| --- | --- |
| `static BlastFilterResult` | `blastForOneBPRepeats(java.lang.String blastAll, java.lang.String database, java.io.File fastaFile)`             This has been tweaked to find 1bp mismatches in a 25mer to a genome. |
| `static void` | `main(java.lang.String[] args)` |
| `static void` | `printDocs()` |
| `void` | `processArgs(java.lang.String[] args)`             This method will process each argument and assign any new varibles |

**Methods inherited from class java.lang.Object**

`equals, getClass, hashCode, notify, notifyAll, toString, wait, wait, wait`

**Constructor Detail**

### TPMapOligoBlastFilter

```
public TPMapOligoBlastFilter(java.lang.String[] args)
```


**Method Detail**

### blastForOneBPRepeats

```
public static BlastFilterResult blastForOneBPRepeats(java.lang.String blastAll,
                                                     java.lang.String database,
                                                     java.io.File fastaFile)
```

:   This has been tweaked to find 1bp mismatches in a 25mer to a genome. No indels.
    Returns the number of matches found in the database, thus a unique oligo would be found once.
    Returns -1 if something went wrong.
    Query id, Subject id, % identity, alignment length, mismatches, gap openings, q. start,q. end, s. start, s. end, e-value, bit score
    0 1 2 3 4 5 6 7 8 9 10 11

---


### processArgs

```
public void processArgs(java.lang.String[] args)
```

:   This method will process each argument and assign any new varibles

---


### printDocs

```
public static void printDocs()
```

---


### main

```
public static void main(java.lang.String[] args)
```


---


|  |  |  |
| --- | --- | --- |
| **Overview** \| **Package** \| **Class** \| **Use** \| **Tree** \| **Deprecated** \| **Index** \| **Help** | |  |
| **PREV CLASS**   **NEXT CLASS** | **FRAMES**    **NO FRAMES**     **All Classes** |  |
| SUMMARY: NESTED \| FIELD \| CONSTR \| METHOD | DETAIL: FIELD \| CONSTR \| METHOD |  |


---
